# Probing Slow Earthquakes With Deep Learning

**DOI:** 10.1029/2019GL085870

**Published:** 2020-02-24

**Authors:** Bertrand Rouet‐Leduc, Claudia Hulbert, Ian W. McBrearty, Paul A. Johnson

**Affiliations:** ^1^ Los Alamos National Laboratory, Geophysics Group Los Alamos NM USA; ^2^ Laboratoire de Géologie, Département de Géosciences, École Normale Supérieure, PSL Research University, CNRS UMR Paris France; ^3^ Department of Geophysics Stanford University Stanford CA USA

**Keywords:** tectonic tremor, deep learning, slow earthquakes, machine learning, Cascadia

## Abstract

Slow earthquakes may trigger failure on neighboring locked faults that are stressed sufficiently to break, and slow slip patterns may evolve before a nearby great earthquake. However, even in the clearest cases such as Cascadia, slow earthquakes and associated tremor have only been observed in intermittent and discrete bursts. By training a convolutional neural network to detect known tremor on a single seismic station in Cascadia, we isolate and identify tremor and slip preceding and following known larger slow events. The deep neural network can be used for the detection of quasi‐continuous tremor, providing a proxy that quantifies the slow slip rate. Furthermore, the model trained in Cascadia recognizes tremor in other subduction zones and also along the San Andreas Fault at Parkfield, suggesting a universality of waveform characteristics and source processes, as posited from experiments and theory.

## Key Points

- Deep learning models can recognize tremor on a single seismic station
- New detections of tremor correlate with local geodetic displacement rate and seem to increase weeks to months before slow earthquakes
- A model trained to detect tremor in Cascadia can detect known tremor from Japan and California with no further training

## 1 Introduction

In many subduction zones where megaquakes occur, slow earthquakes take place deep on the subduction interface, perturbing the stress environment of the neighboring and shallower locked zone, and potentially influencing the occurrence of large megathrust earthquakes (Ito et al., 2013; Obara & Kato, 2016). As a result, the relation between slow earthquakes and the locked zone is a topic of intense interest. Tectonic tremor, the noise‐like seismic signature of slow earthquakes emanating from the subduction zone, was discovered relatively recently (Obara, 2002; Rogers & Dragert, 2003). Tremor is identified and located by analyzing the phase correlations of seismic signal envelopes recorded at multiple seismometers (Obara, 2002; Obara & Kato, 2016). Tremor is frequently used as a qualitative proxy for slow slip (Ide, 2012), including in situations where GPS shows no displacement, presumably because deformation at the surface is too small to be detected geodetically (Frank, 2016).

**Figure 1:**
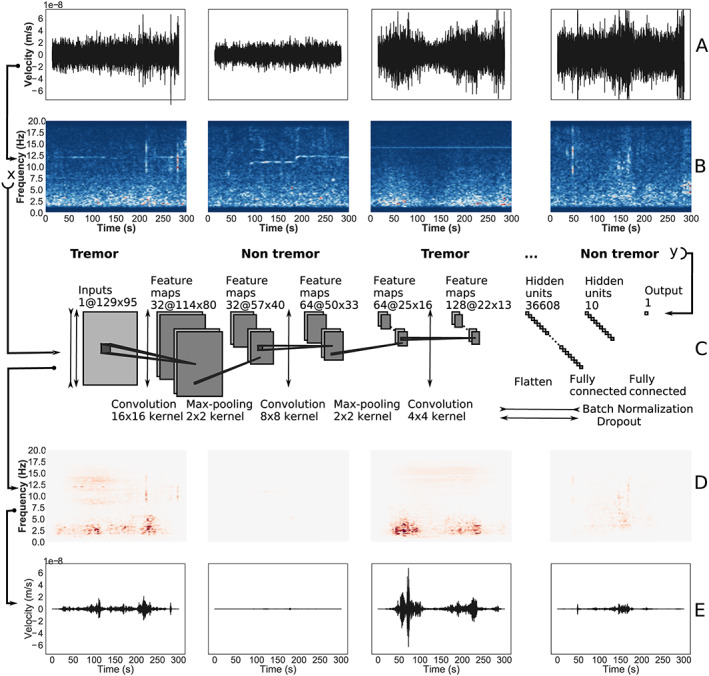
Deep learning tremor. (s) Five‐minute duration, single station (NLLB) seismic signals. (b) Short‐time Fourier transform of the waveforms that are fed as input to the convolutional neural network. (c) Schematic of the CNN and its architecture. The convolutional layers learn representations (features) of tremor while the last dense layers determine detection/no detection of tremor based on the presence of these features in a spectrogram. The model is trained on spectrograms labeled using the PNSN catalog of tremor from southern Vancouver Island from 2009 to 2015. (d) Interpretation of the CNN using Taylor decomposition (Montavon et al., 2017), showing in red which parts of the spectrograms were recognized by the CNN as characteristic of tremor. (e) Reconstruction of the waveforms from only the portions of the spectrograms recognized as tremor according to the CNN and its interpretation.

In an effort to characterize tremor from seismic noise and probe the relationship between tremor and slip (Figure 1), we use a method based on convolutional neural networks (CNNs), a type of deep learning algorithm. CNNs are at the core of recent dramatic advances in computer vision, natural language processing, and recommender systems (Balestriero & Baraniuk, 2018; Ioffe & Szegedy, 2015; Kingma & Ba, 2014; Krizhevsky et al., 2012; LeCun et al., 2015; Nair & Hinton, 2010; Srivastava et al., 2014; Silver et al., 2016, 2018). The use of CNNs to recognize tremor can be viewed as a deep learning extension of template matching methods (Frank et al., 2014; Gibbons & Ringdal, 2006), where the deep learning model automatically determines which time frequency patterns to use, effectively learning something akin to a set of templates that are more general representations of tremor than hand‐crafted templates. Tasked with recognizing tremor from portions of single station seismic data, the convolutional layers learn to represent inputs as a collection of simpler characteristic features. These features are then fed to the dense layers of the CNN, which learns to classify whether a portion of seismic data contains tremor or not based on this transformed representation.

## 2 Results

### 2.1 Deep Learning Tremor

**Figure 2:**
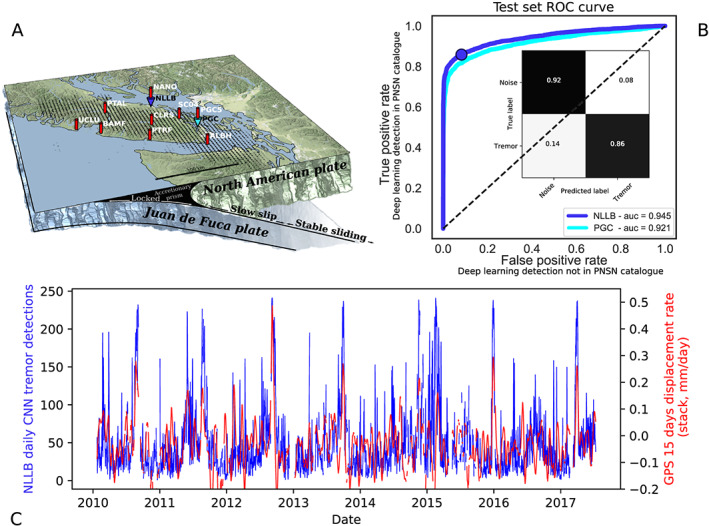
Deep learning detections continuously tracks geodetic slow slip displacement rate and generalizes to nearby stations. (a) Map of Vancouver Island. Slow slip and tremor originate from ductile portions of the interface, downdip from the locked zone where a megathrust earthquake is anticipated. Seismic stations NLLB and PGC as well as the GPS stations used here are noted by colored arrows. (b) The area under the receiving operating characteristic (ROC) curve and the confusion matrix. The ROC curve shows the true (deep learning detection of cataloged event) and false (deep learning detection of a possible but uncataloged event) positive rates as the threshold of classification of the model is varied. A model that reproduces the catalog exactly would yield a point in the upper left corner or coordinate (0,1) of the ROC space. The inset shows the confusion matrix, indicating the fraction of classified noise and tremor by the deep learning model compared to the labels from the multistation catalog, for a model with a threshold of 0.5. Most tremor cataloged using multistation cross correlations are identified on a single station by the neural network. Other signals such as earthquakes, teleseisms, cultural noise and microseisms are easily distinguished from tremor by the model (see also Figure S2 in the supporting information). (c) Blue: Daily detection rate of the deep learning model on seismic data from the NLLB station. Red: 15 days average of the displacement rate, from the stack of the GPS stations in red on the map.

The CNN model is trained on seismic data from the Canadian National Seismograph Network (CNSN) (Canadian National Seismograph Network, 1989). Figure 2a shows the area analyzed: Vancouver Island on the North American Plate and the subducting Juan de Fuca plate, with a schematic of the locked and slowly slipping portions of the downgoing slab. Our ground truth for the initial training of the neural network is a tremor catalog from the Pacific Northwest Seismic Network (PNSN). The catalog was constructed using a correlation‐based tremor identification method (multistation, based upon 5‐min envelope correlation) (Wech & Creager, 2008) from the southern portion of Vancouver Island between October 2009 and July 2017. We build our database from 5‐min portions of single‐station and single‐channel seismograms. For every tremor event in the PNSN catalog, we record the corresponding 5‐min single‐station horizontal component waveform (channel E), and label it as containing tremor. For the nontremor examples we randomly sample the seismic data on days where no tremor was identified in the PNSN catalog. This results in about 47,500 time windows of 5‐min single‐station waveforms labeled as containing tremor, and 47,500 windows of 5‐min single‐station waveforms labeled as *not* containing tremor. In order to leverage the ability of CNNs to extract information from images, instead of feeding raw waveforms to the CNN, we first compute the short‐time Fourier transform (STFT) of the 5‐min portions of data (Figure 1b). The original 5‐min waveforms containing 12,000 data points are converted into spectrograms over the 5‐min interval. These steps result in our database of 95,000 labeled ‘tremor’ and ‘absence of tremor’ examples (Figures 1D and 1E).

We split our database of 95,000 time (frequency) windows into two contiguous portions for training and testing: the first 80% for training and the last 20% for validation (10%) and testing (10%). In other words, all the examples from the end of 2009 to the end of 2015 are used to train the model, the examples from the end of 2015 to the end of 2016 are used for validation, and the examples from the end of 2016 to the end of 2017 are used to test and assess the model. The split of training data into contiguous pieces is of paramount importance for time series in general and for this problem in particular. For instance, applying a random train/test split would assign pieces of any tremor burst longer than a few minutes to both the training and the testing set, making the problem dramatically easier—the CNN would only have to memorize (i.e., overfit) the examples it sees in training.

The architecture of the CNN consists of three convolutional layers and one fully connected hidden layer (see Figure 1 and Text S1 in the supporting information for details). At the end of the training procedure (Figure S1 in the supporting information) the model is fixed and applied to the testing set (the last year of data never analyzed before), to assess how well it generalizes to new examples of tremor.

The CNN outputs an empirical probability that a portion of waveform transformed into the time‐frequency domain contains tremor. The performance of the model, measured through the ROC‐AUC metric (for Area Under Curve of Receiver Operating Characteristic), is shown in Figure 2. The ROC curve is generated by plotting true positive rate of the model on the y axis versus the false positive rate on the x axis at progressively larger threshold settings. The further the curve is from the diagonal line and closer to the upper left corner, the better the model is at discriminating between positives and negatives. Lowering the classification threshold classifies more items as positive, thus increasing both false positives and true positives. For our purposes, true positives are cataloged events detected on a single station by our deep learning model. False positives are detections from our model of possible events that were not cataloged (see Figures 2c, S2, and S4 for arguments that these additional detections are real, as shown by the improved mapping to geodetic displacement and the possible acceleration of new detections before large slow slips). Figure 2b shows the model performance on the test set (last year of the labeled database, 2017) according to the ROC curve: with a ROC‐AUC score of 0.945, the model performs well in discriminating between positives (cataloged events) and negatives (waveforms not cataloged, and on days with no cataloged events). The confusion matrix in inset shows the fraction of classified noise and tremor compared to the actual labels, for a model with a default threshold of 0.5, which is the threshold used during the training phase of the neural network to fit the training portion of the catalog.

### 2.2 Tremorness

Given continuous seismic spectral data as input, the deep learning model outputs an empirical probability that the seismic data contains tectonic tremor. We term this empirical probability “tremorness.” We showed in previous work that the energy of continuously recorded low‐amplitude seismic waves track the smoothed GPS displacement rate well at long timescales ( 30+ days) and short timescales (1 hr) (Rouet‐Leduc et al., 2019). The energy‐GPS correlation may suggest that tremor is emitted continuously or quasi‐continuously at the plate interface from its slowly slipping portion (Figure 2a). In contrast, cataloged tremor has been shown to be intermittent (Wech & Bartlow, 2014). Using the deep CNN trained to recognize cataloged tremor, we find strong evidence that tremor is emitted at least quasi‐continuously, and is a quantitative proxy for geodetic slow slip.

Figure 2c shows that the daily tremor detection rate of our deep learning model (tremorness of the seismic data from the NLLB station greater than 0.5) tracks the colocated SW GPS displacement rate, even at small displacement rates. In between the peaks of slow slip rate, the deep learning model detects 10 to 50 tremor events on any given day (a 3% to 17% detection rate, compared with 0.8% in Michigan, for example, where no slow slip occurs, the actual false positive rate of our model, see Figure S2). Smaller peaks of tremors (in the 50 to 150 events a day) that go undetected in the multistation method map to smaller peaks in the 0.0‐ to 0.2‐mm/day range in the average GPS displacement rate (see Figure S4), further demonstrating the existence of numerous slip events in between the known large slow slip events (Frank, 2016). If we consider a detection threshold of tremor for a tremorness (CNN output) above 0.5 empirical probability, the default classifier built by the deep learning model, we detect more than 130,000 5‐min waveforms containing tremor events, close to 3 times the number contained in the catalog for the same time interval. In particular, we observe numerous detections and an increase in detections several weeks before the large slow slip events, as seen on Figure 2c and shown in the inset of Figure S2.

We note that our neural network has been trained to reproduce the catalog as well as it could using a single seismic station. During the training of the CNN, the decision boundary between tremor and nontremor is at 0.5. This threshold of 0.5 therefore corresponds to the neural network fitting as best as it can the training portion of the tremor catalog, based on the partial information it has (single‐station seismic data). This corresponds to a threshold of 0.5 on the ROC curve on Figure 2b. The deep learning model of tremor is therefore conservatively trained and the additional events it detects do not come from lowering the standards of detection compared to the multistation method. This means that the model finds more events than those in the catalog. This finding is not because these events correspond to a lower detection standard, but because according to the spectral features learned on a single station they are as likely to be tremor as the known cataloged events. These events are too weak in amplitude (see Figure S3) to be detected on multiple stations, but based on the temporal evolution of their frequency content, the deep learning model cannot distinguish these newly detected tremor events from the known cataloged events. The stark increase in correlation between our local deep learning detections and local GPS displacement rate (Figures 2c and S4) further shows that these detections are not spurious. We note here that we are not trying to establish a relationship between seismic moment and geodetic moment (Frank & Brodsky, 2019) but simply to give an assessment of the validity of our additional detections.

As a further test we applied the trained model to seismic data measured in a region known to be tectonically and seismically inactive, in this case Michigan, USA (Figure S2). Extremely little tremor is identified in Michigan by our model, supporting that the numerous newly detected tremor events in Cascadia are real. It also indicates that our model is not confused by cultural noise (e.g., train traffic, vehicular traffic, wind farms), meterological noise (Johnson et al., 2019), or teleseisms.

## 3 Discussion

### 3.1 Deep Learning Model Interpretation

Deep learning models are notoriously hard to interpret. However, recent efforts (Baehrens et al., 2010; Montavon et al., 2017) have showed that perturbing the input of deep learning models enables their analysis, to some extent. In our case the network outputs the empirical probability that the input contains tremor, and a Taylor expansion of the model reveals the time‐frequency components the network used to classify the signal as tremor. Figure1d shows examples of such an analysis, applying a Taylor expansion of the neural network with respect to its input pixels (Montavon et al., 2017) to construct a heatmap of time‐frequency components identified used by the deep learning model to identify tremor. Figure1e shows the inverse short‐time Fourier transform of the time‐frequency components identified as tremor by the Taylor analysis. Here, because the Taylor expansion identifies individual time‐frequency components used to identify tremor, we can reconstruct a signal that represents the separation of the tremor signal from the background noise. We caution that this signal extraction method is only partial, as the Taylor expansion only reveals the time‐frequency components that are characteristic of tremor and not components that may be common between tremor and other signals.

The features learned by the convolutional neural network are patterns in the time‐frequency domain, and we posit these features are directly related to the frictional properties of the slip on the interface that emit the signals. We presume that variations in pore pressure, chemistry, or thermal properties may modulate or influence the emitted signal, but the origin of the signal is due to emissions coming from asperities on the fault interface. This is a point we intend to explore further in future work, along with evolution of these features with time (Holtzman et al., 2018).

### 3.2 Universality of Tremor Characteristics

Deep learning models have tremendous expression capabilities, which can lead to overfitting. In the previous section we demonstrated that our network generalizes to new seismic data from the station it was trained on, recorded later in time, suggesting that overfitting is not an issue. A powerful additional test is to see whether the network generalizes to seismic data from another station. Seismic recordings at a given station are a convolution of the source, propagation path and “site amplification” effects, that may vary tremendously over short distances (Aki & Richards, 2002). Generalization to another station would suggest that the network did not only learn the specifics of the seismic data it has been trained on. In Figure 2b we show that our trained network is robust when applied to other seismic stations on Vancouver Island. The network trained on station NLLB from the end of 2009 to the end of 2015 can accurately recognize cataloged tremor on station PGC from 2016 to 2017, 80 km away. This test supports that the network did learn general time‐frequency dynamics that are characteristic of tremor.

**Figure 3:**
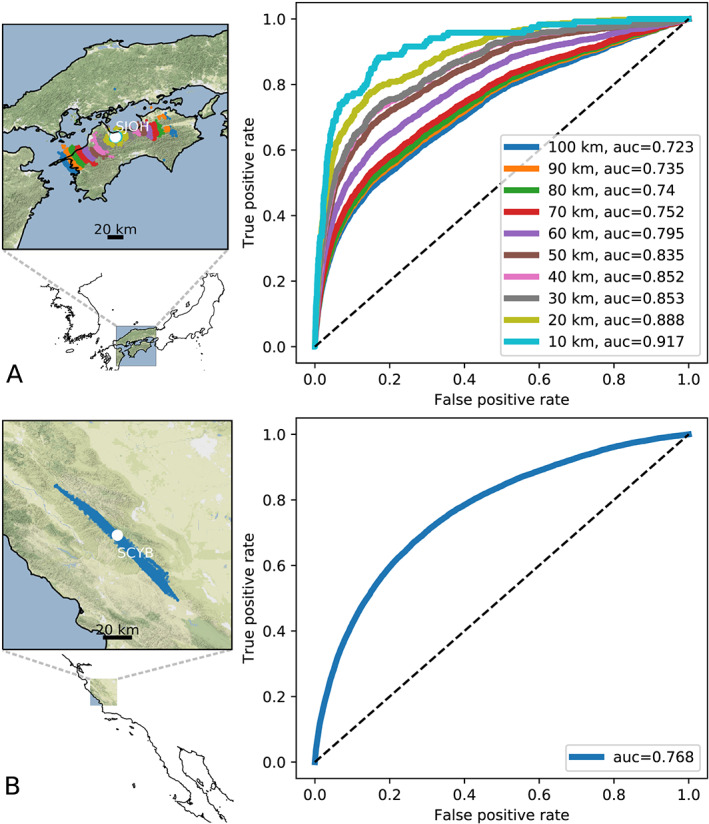
Generalization of the model: The deep neural network model of tremor trained in Cascadia recognizes known tremor in other regions. (left) Maps of cataloged tremor in Japan (A) and California (B). (right) ROC curves showing the accuracy of our model trained in Cascadia at recognizing tremor in Japan (A) and California (B). (a) ROC curves of the deep learning model of tremor exclusively trained in Cascadia and applied to tremor cataloged in Shikoku, Japan (Ide, 2012; Idehara et al., 2014). The performance decreases with distance (colors on maps and ROC curves matching). At short distances ( <20 km) the model has the same performance on Japanese tremor as it does on Cascadia tremor. (b) ROC curves of the deep learning model applied to tremor cataloged on the San Andreas fault (Shelly, 2017). The deep model trained on the Cascadia subduction zone only could not be used to build catalogs on the Parkfield section of the San Andreas transform fault, but its ability to recognize cataloged tremor in most cases underscores the frictional similarity between subduction tremor and tremor at Parkfield.

Furthermore, the model generalizes to other subduction zones and different tectonic environments (Figure 3). Figure 3a shows that cataloged tremor in Shikoku, southern Japan (Ide, 2012; Idehara et al., 2014) (also developed using multistation envelope correlation) is accurately identified on a single station by our deep learning model of tremor trained in Cascadia, with no further training required. Figure 3b shows that known low‐frequency earthquakes (LFEs) emitted by the San Andreas transform fault (Shelly, 2017) —a very different tectonic environment—are also identified by the same deep learning model trained in Cascadia. LFEs on the San Andreas fault have recently been shown to be correlated to slow slip on the deep portion of the fault (Rousset et al., 2019), similar to slow slip in subduction zones. These results show that the time‐frequency dynamics of tremor signals are largely dominated by the source characteristics, because our model is station and even region agnostic.

The ability of our model to recognize tremor in Japan and from the San Andreas fault suggests that the waveform characteristics of tremor are universal. This result is in line with laboratory analysis (Scuderi et al., 2016) and models (Daub et al., 2011)—tremor‐inducing slow slip occurs within frictional conditions that are very similar for a wide variety of faults, and possibly all faults systems.

## 4 Conclusion

We showed that a deep learning model trained in Cascadia accurately detects tectonic tremor on a single station. Trained on cataloged tremor events initially identified by multistation methods, the deep learning model can be used to find many more events. The local detection rate of our deep learning model correlates with the local stacked geodetic displacement rate considerably more so than the initial catalog it has been trained on, giving additional credence to the newly detected events. Deep learning detected tremor seems to arise weeks to months before slow slip is detected geodetically. Trained only in Cascadia, the deep learning model recognizes known tremor from other subduction zones as well as from the deep portion of the San Andreas fault, arguing for the universality of the frictional properties governing slow earthquakes.

## Data and Code Availability

The data used are publicly available and can be found online. The seismic data come from the Canadian National Seismograph Network (Canadian National Seismograph Network, 1989) (www.earthquakescanada.nrcan.gc.ca), and the GPS data come from the Western Canada Deformation Array (WCDA) operated by the Geological Survey of Canada (GSC), preprocessed by the USGS (Murray & Svarc, 2017) (https://earthquake.usgs.gov/monitoring/gps/Pacific_Northwest, NA‐fixed trended data). The known tremor catalog used is available from the Pacific Northwest Seismic Network (https://pnsn.org/tremor/tremor-map-legacy). The work flow described in the Methodology section of the supporting information uses open source software: python and python packages including scikit‐learn (Pedregosa et al., 2011), tensorflow (https://www.tensorflow.org/), keras (https://keras.io/) and obspy (Beyreuther et al., 2010).

## Supporting information



Supporting Information S1

Figure S1

Figure S2

Figure S3

Figure S4
