# Supplementary material for: Probing Slow Earthquakes With Deep Learning
Source: Geophys Res Lett. 2020 Feb 24;47(4):e2019GL085870. doi: 10.1029/2019GL085870 (PMC7375133; doi:10.1029/2019GL085870)
Supplement: Supplementary file 1 — Supporting Information S1 [file GRL-47-e2019GL085870-s001.pdf]

Supplementary for: Probing slow earthquakes with deep learning

Bertrand Rouet-Leduc[1], Claudia Hulbert[1,2], Ian W. McBrearty[1,3], Paul A. Johnson[1]

[1]Los Alamos National Laboratory, Geophysics Group, Los Alamos, New Mexico, USA
[2]Laboratoire de Géologie, Département de Géosciences, École Normale Supérieure, PSL Research University, CNRS UMR 8538, Paris, France
[3]Department of Geophysics, Stanford University, Stanford, California, USA

1 Methodology

In this section we go through a step-by-step description of our deep learning method. We use seismic data from the Canadian National Seismograph Network ("Canadian National Seismograph Network", 1989). The seismic data are recorded or re-sampled at 40 Hz. In order to prepare a database for training a deep convolutional network, we go through the following procedure:

1) We extract each 5 minute portion of seismic data identified as containing tectonic tremor identified in Wech's tremor catalogue(Wech & Creager, 2008), recorded from under Vancouver Island, from 2008 to 2017. This results in 47500 time slices of 5 minutes of the horizontal component for each seismic station considered (NLLB and PGC).

2) In order to include negative examples in the database, we extract 47500 portions of 5 minutes of seismic data for each station, at times randomly picked during days with no tremor activity noted in the catalogue.

3) Each 5 minute time slice of seismic data is corrected for instrument response by dividing the amplitude by the total gain, and bandpassed between 2 and 18 Hz using a 5th order Butterworth filter. Each time slice is then clipped in amplitude between -5×10^{-8} and $+5\times10^{-8}$ m/s because we do not wish to analyze earthquake and other large amplitude signals.

4) Each time slice is converted into its time-frequency domain using a short-time Fourier transform with a bin size of 256. This turns the seismic time series signals comprised of 12001 data points (5 minutes at 40 Hz) into 129×95 spectrograms (129 time and 95 frequency bins).

5) Each 5 minutes spectrogram is placed into a database and is labeled as containing tremor (time slice during catalogued tremor) or no tremor (randomly sampled during a day with no catalogued tremor).

6) The database is split into training, validation, and testing sets, using the years 2008 to 2015, 2015 to 2016, and 2016 to 2017, respectively, for an 80-10-10% contiguous split.

7) We use the Keras and Tensorflow packages to build our neural network (architecture Fig. 1), compiled with an Adam optimizer (Kingma & Ba, 2014).

8) The neural network is trained on the database described above until the performance on the validation set begins to decrease (Fig. S1).

Corresponding author: B. Rouet-Leduc, `bertrandrl@lanl.gov`

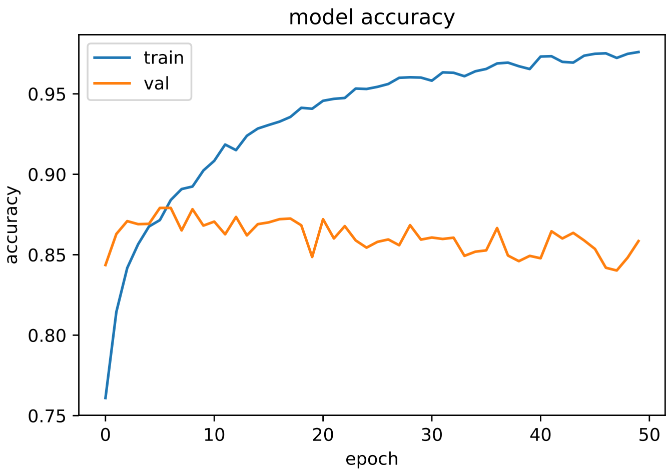

Figure 1. Deep learning tremor. Training is terminated after 4 epochs, when the performance on the validation set begins to decrease, a sign of overfitting. Training, validation, and testing sets are contiguous and separate in time to avoid contamination of the testing set.

9) The final performance of the deep learning model of tremor is assessed on the testing set (Fig. 2, main text). The area under curve metric (AUC) is shown in Fig. 2 in the main text. The best possible prediction would yield a point in the upper left corner or coordinate (0,1) of the ROC space, representing 100% sensitivity (no false negatives) and 100% specificity (no false positives). A random guess would lie along the diagonal line from the left bottom to the top right corners. The ROC shows very good performance of the model, *i.e.* is it very performant in reproducing the multi-station catalogue with only information from a single station.

For the results of Fig. 3 in the main text, steps 1 through 5 are repeated for seismic data and tremor catalogues from Shikoku, Japan (Ide, 2012; Idehara et al., 2014) and the San Andreas fault near Parkfield, California (Shelly, 2017), using the SJOH hi-net station in Japan and the SCYB station in California.

We note that if the quality of the training catalogue from the PNSN improved over time, our model is trained with the earlier, lower quality portion of it, making its task harder.

2 Deep learning tremor

In recent, well known applications such as image recognition and games (Go, chess, video games), humans were outperformed by deep neural networks (Silver et al., 2016, 2018). They feed off vast amounts of training examples to automatically learn the features to transform raw data, in contrast to more traditional machine learning methods where features are hand-crafted. In CNNs, these features (filters) are learned by the convolutional layers of the network (Krizhevsky et al., 2012), and then fed to a multilayer perceptron (also called dense layers), that is the actual model that gives a prediction based on the activation of the convolution filters (see Fig. 1).

Intuitively, the convolutional layers of a CNN are trained to find a set of 'physical transformations' that turn a complicated image into simpler and more general features. In image recognition, convolutional layers can be trained to make a complicated image of a subject evolve into characteristics unique to it, e.g., wheels of a vehicle or whiskers

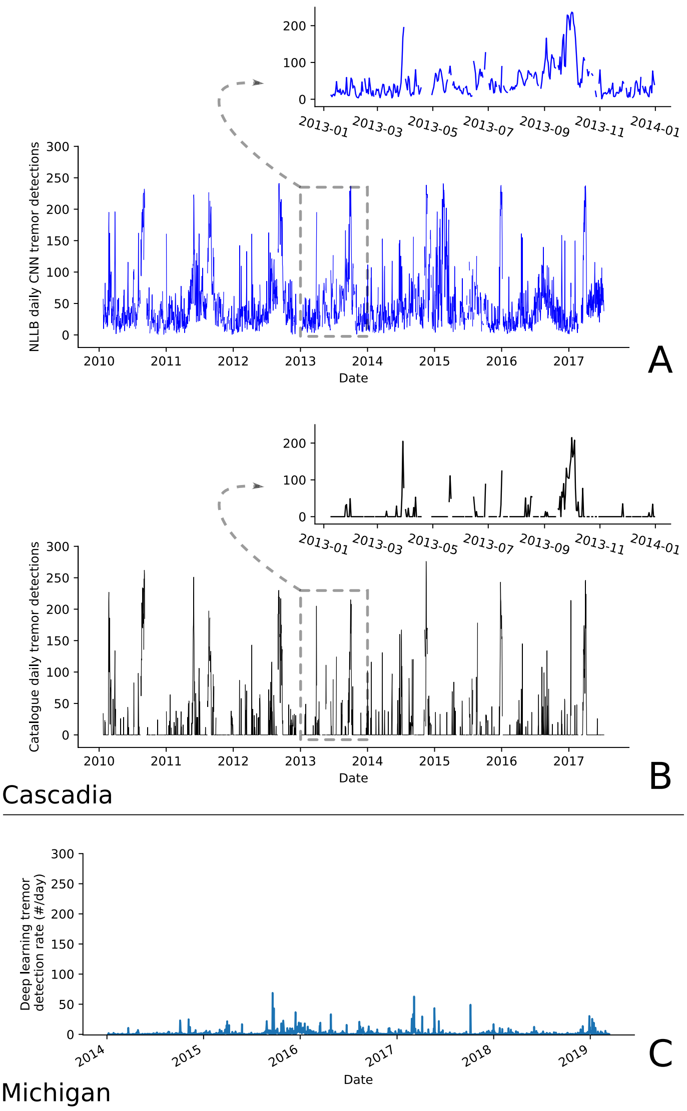

Figure 2. The single station deep learning model detects about three times as many tremor events, without compromising its false detection rate. (A) Tremor event detection rate of the multi-station method (Wech & Creager, 2008) initially used to train the deep learning model. **(B)** Event detection rate of our deep learning model in Cascadia (NLLB station). Tremor events are continuously detected, revealing numerous smaller slow slips. The inset in A and B show an example on tremor detection possibly accelerating before large slip events that is apparent only with the deep learning detection, and much larger than the false positive rate. **(C)** Event detection rate of our deep learning model in Michigan (G40A) where there exists no known tremor, showing a false positive detection rate of 0.008 (0.8%) an extremely small value comparable to regular earthquake detection methods, meaning our model is not mistaking noise sources for tremor, and that the training catalog is not contaminated by such noise sources.

of a cat. Those simpler activated shapes are then fed to the dense layers that relate these unique features to the subject of the image (e.g., whiskers → cat).

A more mathematical interpretation of deep CNNs is given realizing that the convolution operator learned by a filter and applied at every point of the input image is effectively a local linear combination of neighboring points of the input. Pooling and ac-

tivation operations (convex operations) are applied to the convolution operations, effectively making a deep CNN a form of high-dimensional composition of spline operators (Balestriero & Baraniuk, 2018).

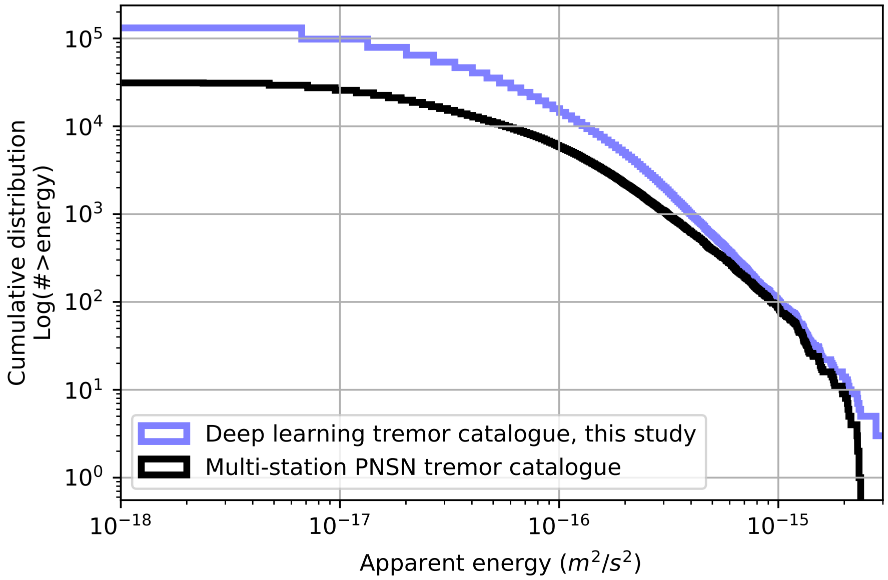

Figure 3. Newly detected events by the CNN model are too weak to reach several stations. Cumulative distribution of apparent event amplitude, given as the square of the 60-40% range of the seismic velocity during the event at station NLLB. Black: known tremor events from the PNSN (Wech & Creager, 2008) catalogue. Blue: events detected by our deep learning model (tremorness>0.5).

The architecture of our CNN consists of three convolutional layers (with 32, 64, and 128 filters respectively, and each with a ReLu activation function (Nair & Hinton, 2010)) and one fully connected hidden layer (with 10 neurons and a sigmoid activation function), itself fully connected to the last visible layer, that has a sigmoid activation function as well (see Fig 1 of the main text). A max-pooling operation is applied after each convolution. Each batch is normalized (Ioffe & Szegedy, 2015) and a 5% dropout (Srivastava et al., 2014) is applied after each convolution layer such that at each model training iteration 5% of the input is randomly set to zero, preventing overfitting by regularizing the model.

Training the CNN is accomplished by backpropagation, using a variation of gradient descent, Adam optimization (Kingma & Ba, 2014). The weights of the CNN are updated iteratively on batches of data from the training set such that at each iteration the weights are adjusted to minimize the error of the model, following the gradient of the error with respect to the weights.

We train our CNN in successive batches of 500 examples: the training set is shown to the CNN in batches of 500 examples, and the CNN is updated after seeing each batch (using gradient descent and backpropagation as described above). Using a standard procedure, the model is trained until its performance on the validation set reaches a maximum. Further training would see the training performance climbing further while the validation score would drop, a telltale sign of overfitting.

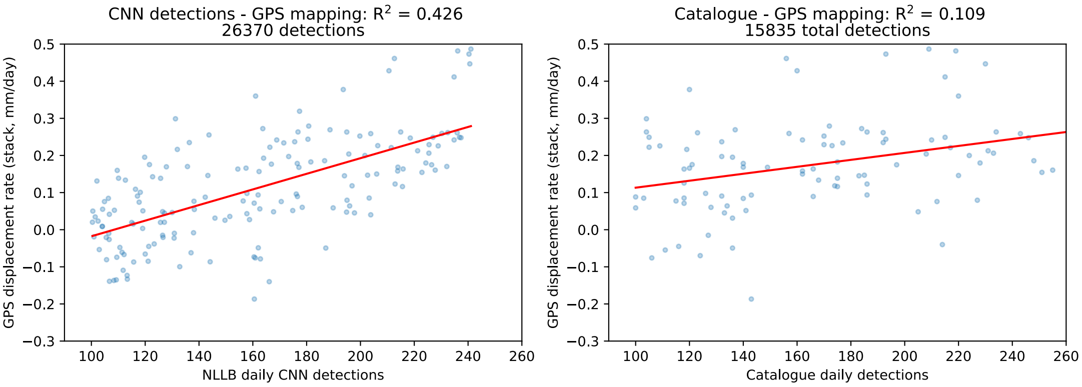

Figure 4. Deep learning tremor detections are a proxy for slow slip. Left: NLLB station daily deep learning tremor detections peaks (above 100/day). Right: daily catalogue peaks (above 100/day) versus GPS displacement rate (15 days average, NANO station), with linear fit (red line) overlayed. The deep learning tremor detections show a better mapping ($R^2 = 0.426$) to geodetic displacement rate (from stacking GPS displacement rates from stations on Fig. 2) compared with the catalogued tremor ($R^2 = 0.109$), giving additional evidence that the new detections are real.

3 Tremorness as a quantitative proxy for slow slip

In order to measure the displacement rate presumed to be taking place at the downdip transitional section of the subduction zone where slow slip occurs, we use as a proxy the data from nearby GPS stations from the Western Canada Deformation Array, preprocessed by the United States Geological Survey (Murray & Svarc, 2017). We take the total horizontal displacement (east/west + north/south GPS components), such that a negative displacement is in the direction of the subduction (northeast), and a positive displacement is in the direction of the slip (southwest). The GPS time series are very noisy at short time scales and the denoised displacement rate is calculated as the slope of a least squares linear regression over 15 days for Figs. 2 and S4.

In Fig. S4 we show that the daily tremorness(in red), (*i.e.* the daily moving average of the output from our deep learning model, and also the empirical mean tremor content detected by our model) maps to GPS displacement rate (in blue), whereas the multi-station catalogue rate doesn't. Tremorness provides a quantitative and precise proxy for slow slip.

References

Balestriero, R., & Baraniuk. (2018, jul). A Spline Theory of Deep Learning. *Proceedings of the 35th International Conference on Machine Learning*, 374–383. Retrieved from `http://proceedings.mlr.press/v80/balestriero18b.html` doi: 10.1017/CCOL0521824737.025

Canadian National Seismograph Network. (1989). *Geological Survey of Canada.* doi: 10.7914/SN/CN

Ide, S. (2012, mar). Variety and spatial heterogeneity of tectonic tremor worldwide. *Journal of Geophysical Research: Solid Earth*, *117*(B3). Retrieved from `http://doi.wiley.com/10.1029/2011JB008840` doi: 10.1029/2011JB008840

Idehara, K., Yabe, S., & Ide, S. (2014, dec). Regional and global variations in the temporal clustering of tectonic tremor activity New Perspective of Subduction Zone Earthquake. *Earth, Planets and Space*, *66*(1), 66. Retrieved from

https://earth-planets-space.springeropen.com/articles/10.1186/1880-5981-66-66 doi: 10.1186/1880-5981-66-66

Ioffe, S., & Szegedy, C. (2015, feb). Batch Normalization: Accelerating Deep Network Training by Reducing Internal Covariate Shift. *arXiv preprint*. Retrieved from http://arxiv.org/abs/1502.03167

Kingma, D. P., & Ba, J. (2014, dec). Adam: A Method for Stochastic Optimization. *arXiv preprint*. Retrieved from http://arxiv.org/abs/1412.6980

Krizhevsky, A., Sutskever, I., & Hinton, G. E. (2012, jan). ImageNet Classification with Deep Convolutional Neural Networks. In *Advances in neural information processing systems* (pp. 1097–1105). Curran Associates, Inc. Retrieved from http://papers.nips.cc/paper/4824-imagenet-classification-wpapers3://publication/uuid/1ECF396A-CEDA-45CD-9A9F-03344449DA2A

Murray, J. R., & Svarc, J. (2017). Global Positioning System Data Collection, Processing, and Analysis Conducted by the U.S. Geological Survey Earthquake Hazards Program. *Seismological Research Letters*, *88*(3), 916.

Nair, V., & Hinton, G. E. (2010). Rectified linear units improve restricted boltzmann machines. In *Proceedings of the 27th international conference on machine learning (icml-10)* (pp. 807–814).

Shelly, D. R. (2017, may). A 15 year catalog of more than 1 million low-frequency earthquakes: Tracking tremor and slip along the deep San Andreas Fault. *Journal of Geophysical Research: Solid Earth*, *122*(5), 3739–3753. Retrieved from http://doi.wiley.com/10.1002/2017JB014047 doi: 10.1002/2017JB014047

Silver, D., Huang, A., Maddison, C. J., Guez, A., Sifre, L., van den Driessche, G., ... Hassabis, D. (2016, jan). Mastering the game of Go with deep neural networks and tree search. *Nature*, *529*(7587), 484–489. Retrieved from http://www.nature.com/articles/nature16961 doi: 10.1038/nature16961

Silver, D., Hubert, T., Schrittwieser, J., Antonoglou, I., Lai, M., Guez, A., ... Hassabis, D. (2018). A general reinforcement learning algorithm that masters chess, shogi, and Go through self-play. *Science*, *362*(6419), 1140–1144. Retrieved from http://science.sciencemag.org/content/362/6419/1140 doi: 10.1126/science.aar6404

Srivastava, N., Hinton, G., Krizhevsky, A., Sutskever, I., & Salakhutdinov, R. (2014). Dropout: A Simple Way to Prevent Neural Networks from Overfitting. *Journal of Machine Learning Research*, *15*, 1929–1958. Retrieved from http://jmlr.org/papers/v15/srivastava14a.html

Wech, A. G., & Creager, K. C. (2008). Automated detection and location of Cascadia tremor. *Geophysical Research Letters*, *35*(20).
