# Supplementary figures and images for: Probing Slow Earthquakes With Deep Learning
Source: Geophys Res Lett. 2020 Feb 24;47(4):e2019GL085870. doi: 10.1029/2019GL085870 (PMC7375133; doi:10.1029/2019GL085870)

model accuracy

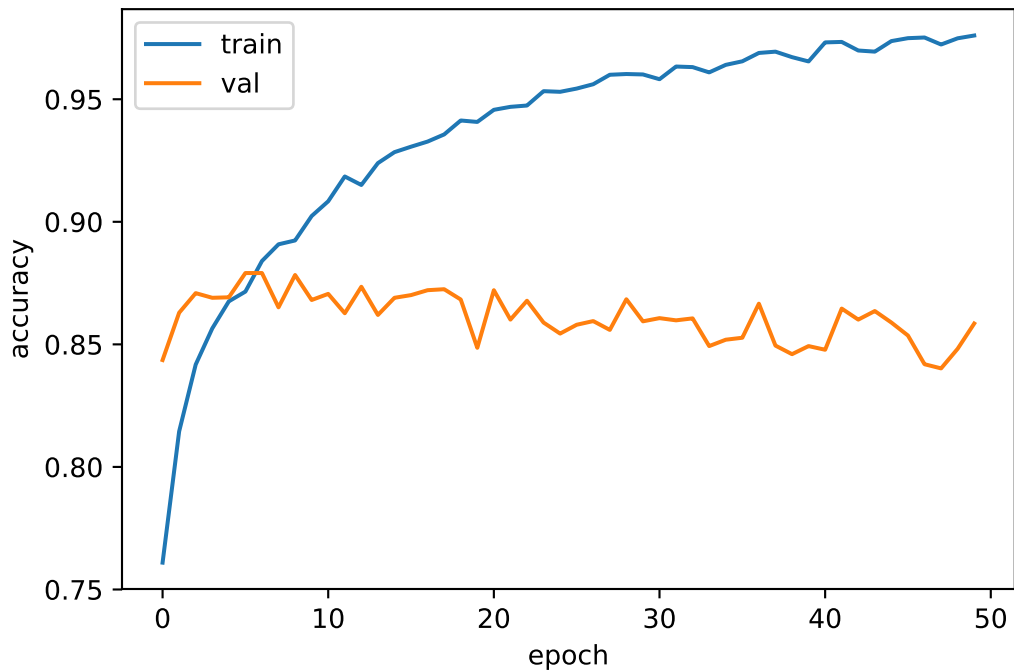

Supplement: Supplementary file 2 — Figure S1 [file GRL-47-e2019GL085870-s002.pdf]

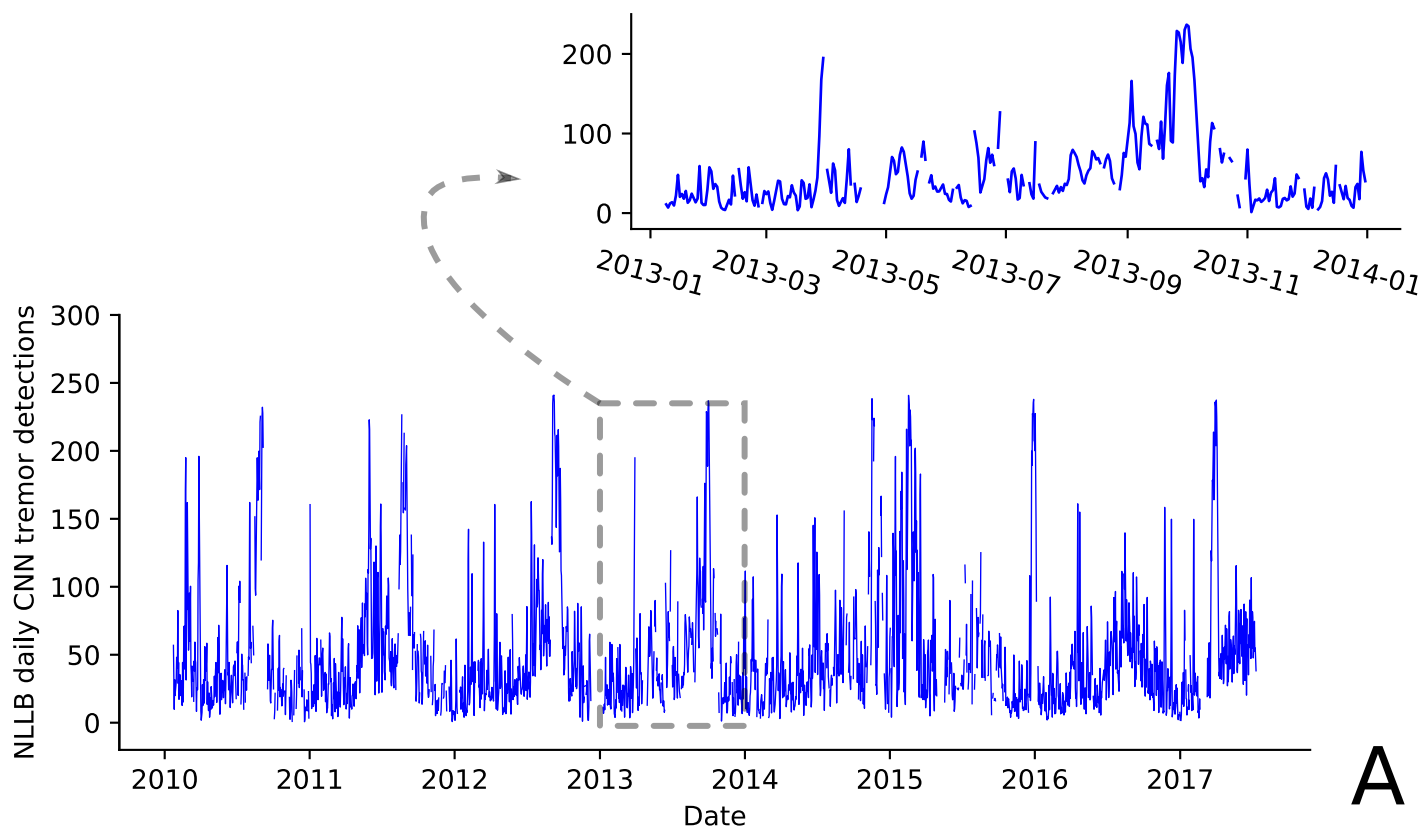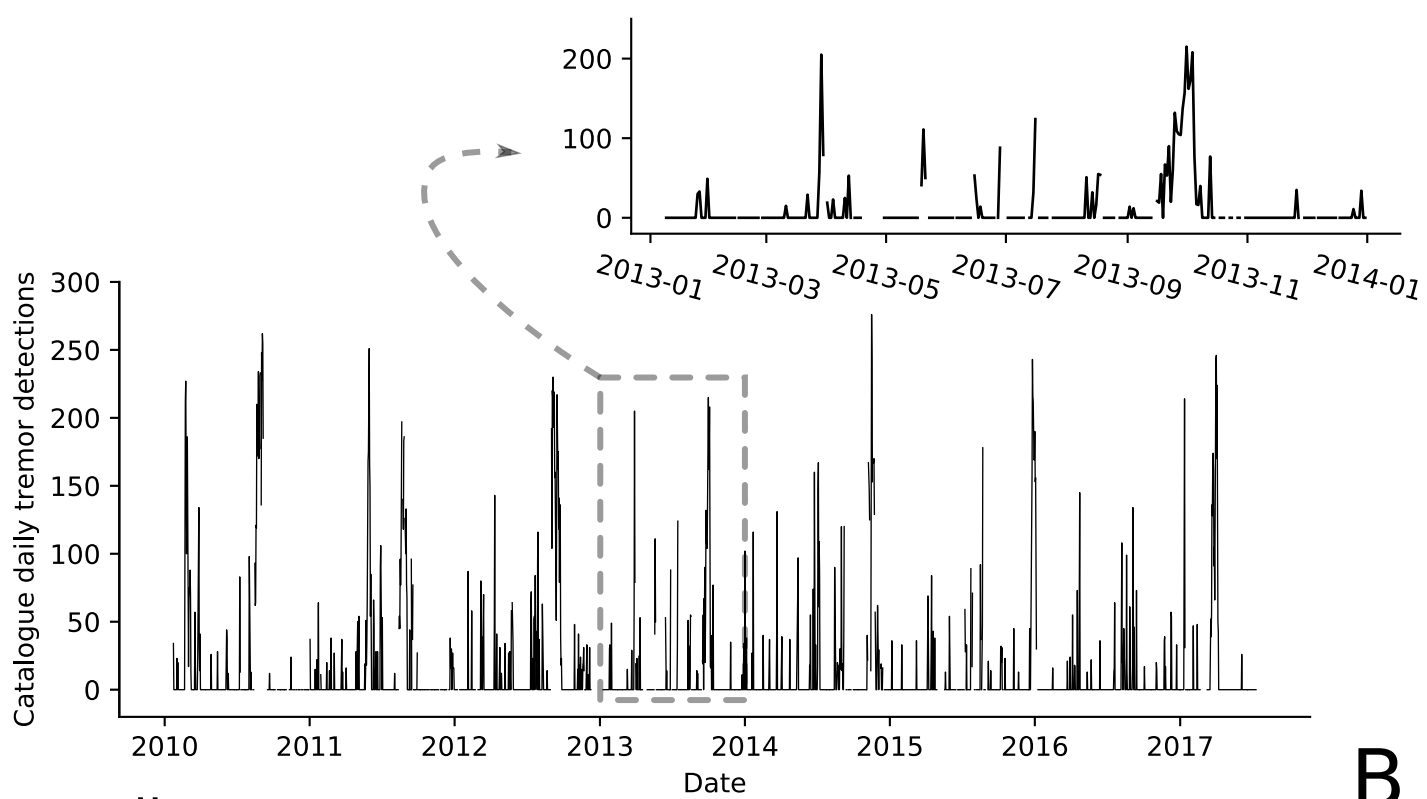

Cascadia

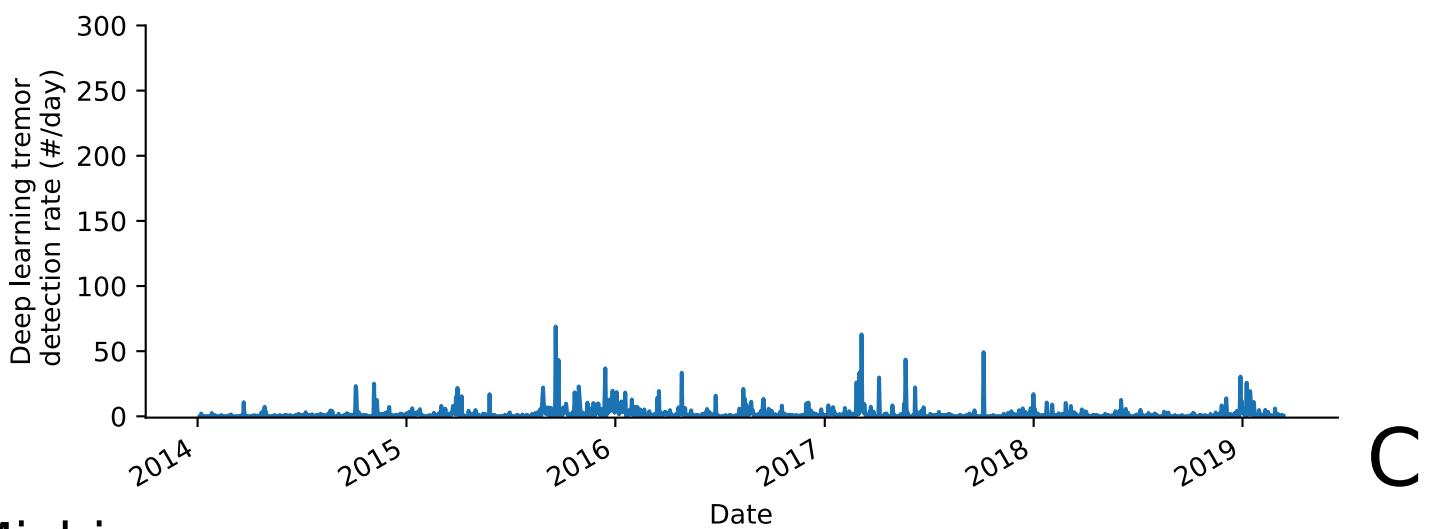

Michigan

Supplement: Supplementary file 3 — Figure S2 [file GRL-47-e2019GL085870-s003.pdf]

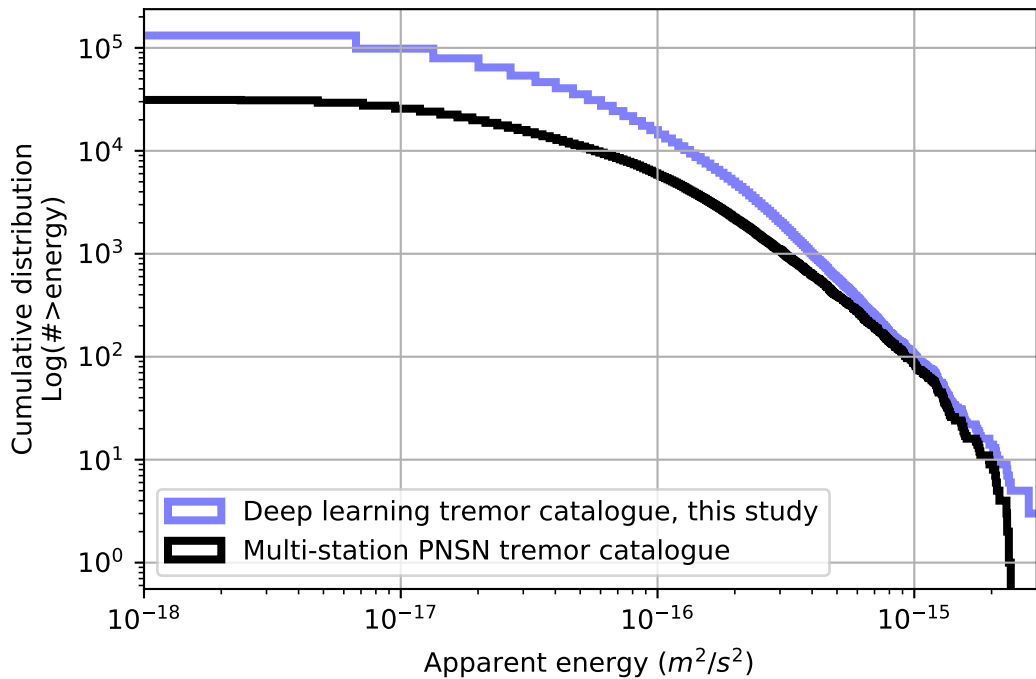

Supplement: Supplementary file 4 — Figure S3 [file GRL-47-e2019GL085870-s004.pdf]

CNN detections - GPS mapping:  $R^2 = 0.426$   
26370 detections

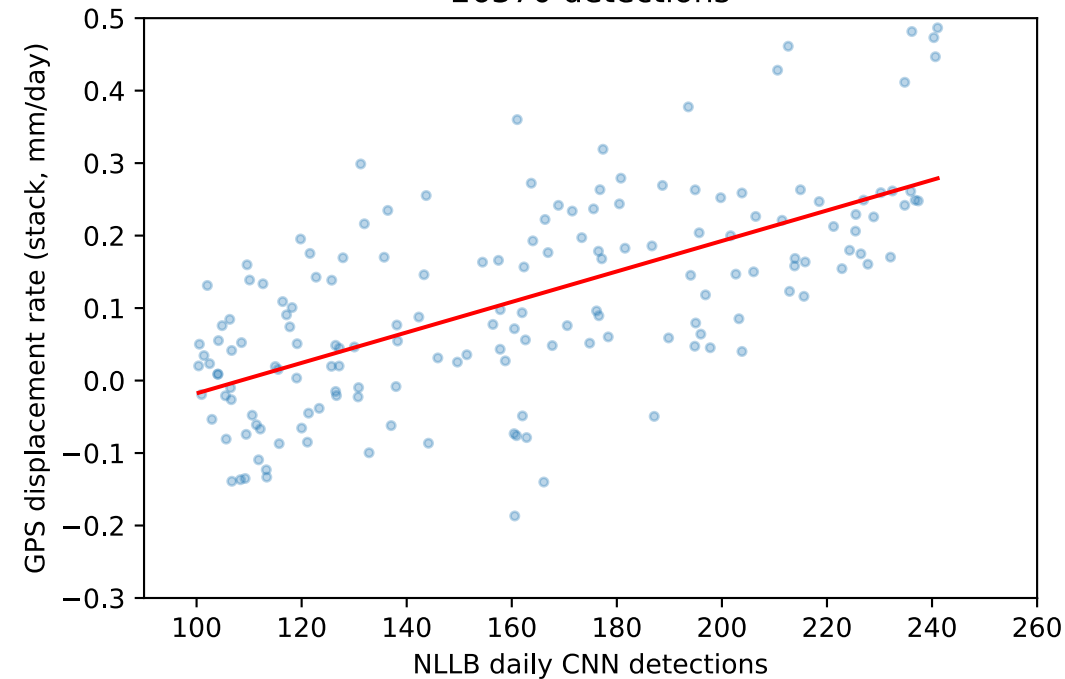

Catalogue - GPS mapping:  $R^2 = 0.109$   
15835 total detections

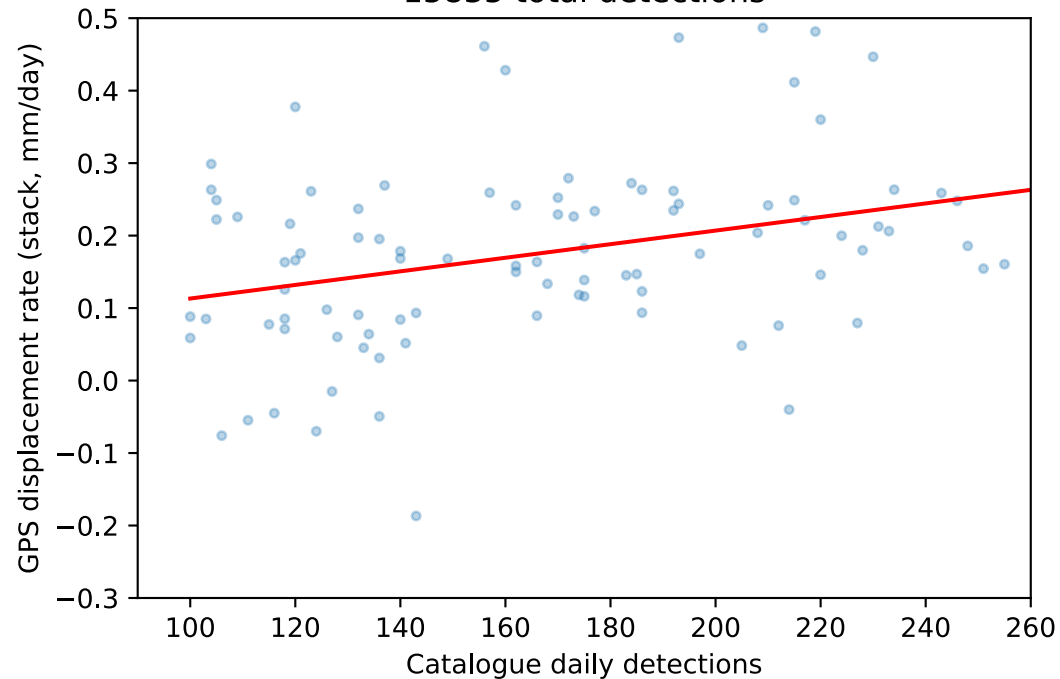

Supplement: Supplementary file 5 — Figure S4 [file GRL-47-e2019GL085870-s005.pdf]
